# Preparation of Polymyxin B-Functionalized Cryogels for Efficient Endotoxin Removal from Protein Solutions

**DOI:** 10.3390/gels11060402

**Published:** 2025-05-28

**Authors:** Peiji Liu, Hong Lin, Jingxue Wang

**Affiliations:** College of Food Science and Engineering, Ocean University of China, Huangdao Campus, Qingdao 266400, China; liupeiji@stu.ouc.edu.cn (P.L.); linhong@ouc.edu.cn (H.L.)

**Keywords:** cryogel, endotoxin, polymyxin B, protein

## Abstract

To address the limitations of traditional endotoxin adsorbents, which exhibit poor endotoxin removal efficiency and low sample recovery when processing high-concentration samples, a novel cryogel, CG(HEMA-co-AM), based on acrylamide (AM) and hydroxyethyl methacrylate (HEMA) as the second monomer, was successfully designed and synthesized. After optimizing the epoxidation and polymyxin B (PMB) conjugation processes, leading to the successful preparation of the functionalized cryogel CG(HEMA-co-AM)@ECH@PMB, flow-through experiments showed that in Tris-HCl buffer at a flow rate of 6 mL/min, the endotoxin removal efficiency reached 99.82%, with a maximum adsorption capacity of 1408.38 EU/mg. In a complex protein system containing BSA, HSA, Hb, LYS, and OVA (each at 10 mg/mL), the maximum endotoxin removal efficiency was 99.62%. Further investigations revealed that pH and ionic strength critically influenced the regulation of hydrophobic and electrostatic interactions, thus significantly affecting the endotoxin adsorption efficiency. Additionally, weakly hydrophobic and basic lysozyme exhibited significantly higher recovery rates compared to strongly hydrophobic and acidic proteins such as BSA and OVA. This functionalized cryogel integrates a high adsorption capacity with excellent specificity and features a macroporous structure suitable for dynamic chromatographic separation. It offers a novel, reusable adsorbent material for endotoxin removal in protein preparation, biopharmaceutical production, and clinical blood purification applications.

## 1. Introduction

In the biotechnology industry, Gram-negative bacteria are extensively used for the production of bioproducts such as peptides and proteins; consequently, these products often contain substantial residual endotoxin [1,2]. Moreover, even when starting materials are initially endotoxin-free, pyrogen contamination may be introduced during manufacturing or operational processes [3,4]. Therefore, the safety and efficacy of protein therapeutics, recombinant proteins, and cell-culture-derived products represent core considerations in research and production.

Endotoxin, also known as lipopolysaccharide (LPS), is a critical component of the Gram-negative bacterial cell envelope [5,6], distinguished by its unique chemical architecture and biological activities. This macromolecular complex comprises lipid A, a core oligosaccharide region, and an O-antigen polysaccharide chain [7,8]. The hydrophilic, negatively charged O-antigen protects bacteria from phagocytic attack and assists in evading host immune clearance [9]. The core oligosaccharide primarily serves as the molecular linker within LPS [10]. Lipid A, the toxic moiety of LPS, embeds within the lipid bilayer of the bacterial cell wall, enhancing membrane stability and releasing endotoxin activity upon bacterial death. Each domain is indispensable for maintaining overall LPS structure and function. Furthermore, LPS exhibits remarkable stability, retaining full biological activity even under extreme conditions of high temperature, strong acid, strong base, or potent oxidants, a resilience that stems from its complex molecular structure and strong interdomain interactions [11,12].

Biologically, endotoxin exerts a broad spectrum of effects. Even at extremely low concentrations, LPS can perturb the host’s thermoregulatory center, inducing fever, leukopenia, microcirculatory disturbances, and systemic inflammatory responses [13,14]. These pathophysiological reactions arise from LPS-host immune system interactions and underscore LPS’s pivotal role during infection. Additionally, LPS stimulates cytokine release and promotes leukocyte recruitment to sites of infection to counter bacterial invasion [15]. However, excessive immune activation may lead to tissue damage and exacerbated inflammation, causing significant host injury [16].

As an essential Gram-negative bacterial cell-wall constituent, endotoxin poses a major safety hazard in medical and biotechnological products. To ensure the safety and efficacy of these products, the fields of bioprocessing and pharmaceutical manufacturing have long focused on developing efficient and reliable endotoxin removal technologies. Continued research and optimization of these decontamination methods are crucial for improving the quality of biopharmaceuticals and medical products. Currently, various physical approaches—including ultrafiltration, solvent extraction, activated-carbon adsorption, affinity adsorption, and chromatographic methods—have attracted significant attention for endotoxin removal [17,18,19]. Kavianpour employed enhanced magnetic nanoparticles (MNPs) as LPS-affinity adsorbents in synergy with a surfactant to achieve highly efficient removal of lipopolysaccharide from rHBsAg [20]. Jandosov utilized biomass-derived nanoporous carbon honeycomb monoliths for the adsorption of environmental lipopolysaccharides from aqueous media [21]. Ling demonstrated that histidine-functionalized polyacrylonitrile-based adsorbents exhibit excellent performance in the simultaneous removal of bacteria and endotoxin from septic blood [22].

Affinity adsorption, as a highly selective separation technique, has demonstrated tremendous potential in biomedical applications, particularly for endotoxin removal [23,24,25]. However, conventional chromatographic media such as synthetic resins and agarose gels often suffer from pore-size limitations, leading to clogging and denaturation when processing high-concentration or complex protein solutions, which in turn results in suboptimal endotoxin clearance and low sample recovery [26,27]. To overcome these challenges, researchers have begun exploring novel adsorbent materials. Among these, cryogels have emerged as a focal point owing to their unique macroporous structures, exceptional permeability, and robust physicochemical stability.

**Figure 1:**
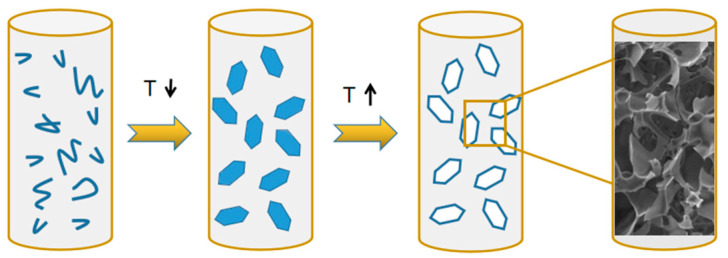
Schematic diagram of the preparation principle of cryogel.

Cryogels are three-dimensional, flexible polymer networks synthesized via freeze–thaw templating techniques [28]. Their fabrication involves complex solution phase separation and ice-templating processes [5,29]. As shown in Figure 1, rapid freezing induces solvent crystallization to form ice, expelling polymer solutes into the interstitial unfrozen microphases. These concentrated solutes subsequently crosslink chemically within the microphases, forming a three-dimensional network intertwined with the ice templates [30,31,32]. Upon removal of ice crystals—commonly by freeze-drying—a macroporous cryogel is obtained, featuring a 3D network of large, interconnected pores that confer excellent chemical and structural stability [33,34].

Cryogels produced by this approach exhibit high porosity and large specific surface area, providing substantial adsorption capacity and enhanced interaction with biomolecules, thereby improving biocompatibility and separation efficiency [35,36]. Nonetheless, unmodified cryogels lack molecular specificity, necessitating incorporation of affinity ligands. Covalent grafting of functional groups onto the cryogel backbone is critical to stably immobilize ligands and enable rapid, selective biomolecular capture and purification [37].

Polymyxin B, a cyclic peptide antibiotic derived from Paenibacillus polymyxa fermentation [38], occupies a prominent place in medicine due to its potent antibacterial activity and LPS-neutralizing capability. Its bactericidal mechanism involves electrostatic interaction with bacterial membrane phospholipids, disrupting membrane integrity and causing intracellular leakage, ultimately effecting bacterial death [39]. PMB shows strong activity against Gram-negative pathogens such as Pseudomonas aeruginosa, Escherichia coli, Klebsiella pneumoniae, and Haemophilus influenzae. Additionally, PMB binds to specific anionic sites on the bacterial cell wall, further compromising structural integrity and accelerating bacteriolysis [40]. In endotoxin adsorption applications, PMB’s cationic amino groups and hydrophobic alkyl chains enable dual electrostatic and hydrophobic interactions with LPS, making PMB an ideal affinity ligand.

In this study, hydroxyethyl methacrylate was selected as the second monomer, and epichlorohydrin (ECH) was utilized to introduce epoxy functionalities into the cryogel network, with process optimization to enhance epoxy activation efficiency. The objective was to develop a novel, high-efficiency endotoxin adsorbent by incorporating PMB as a specific affinity ligand into the optimized cryogel scaffold. To evaluate the performance of CG(HEMA-co-AM)@ECH@PMB, endotoxin removal assays were conducted in Tris-HCl buffer and in five different protein solutions. By systematically varying the experimental parameters (pH, flow rate, contact time), we monitored the dynamic adsorption and desorption behaviors of LPS and proteins, elucidated interaction mechanisms among endotoxin, cryogel, and co-present proteins, and assessed the application efficacy in protein samples. Successful development of this material is expected to provide a novel solution for safe recombinant protein and peptide production and to advance downstream purification technologies in the biopharmaceutical field.

## 2. Results and Discussion

### 2.1. Preparation and Characterization of CG(AM)

**Figure 2:**
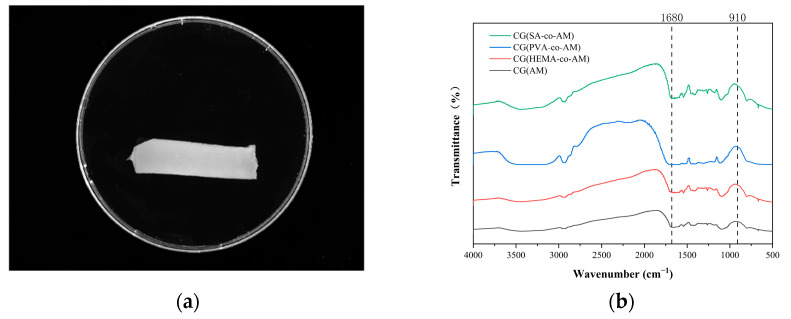
(**a**) CG(AM) surface topography; (**b**) FT-IR spectrum of CG(AM).

The cryogel CG(AM) was synthesized by a low-temperature polymerization method. The surface of the cryogel appeared smooth and uniform (Figure 2a). According to the Fourier transform infrared (FT-IR) spectroscopy results (Figure 2b), in the wavenumber range of 1650–1750 cm^−1^, the observed complex band was composed of multiple overlapping C=O characteristic peaks. Notably, the peak at 1680 cm^−1^ corresponds to the stretching vibration mode of the C=O group in the -CONH_2_ moiety. Although this characteristic band partially overlaps with other C=O bands in frequency, spectral deconvolution allowed for accurate identification and differentiation of this specific signal [41].

**Figure 3:**
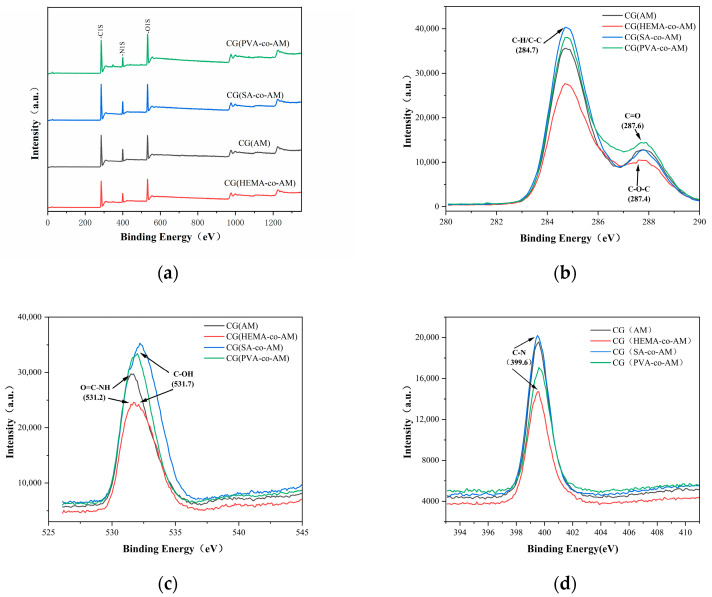
XPS characterization results of cryogels: (**a**) XPS general spectrum; (**b**) C1s characterization; (**c**) O1s characterization; (**d**) N1s characterization.

The absorption band at 910 cm^−1^ indicates the presence of epoxy groups within the cryogel, confirming the successful incorporation of epoxy functionalities via copolymerization [42]. This was further supported by the XPS results shown in Figure 3, validating the successful synthesis of epoxy-group-containing acrylamide cryogel CG(AM). The epoxy-group density, determined by the sodium thiosulfate titration method, was measured to be 22.31 μmol/g. To enhance the subsequent PMB coupling efficiency of CG(AM) for high-efficiency endotoxin adsorption, it was necessary to further increase the epoxy-group density of the cryogel.

### 2.2. Preparation and Characterization Studies of CG(HEMA-co-AM)

During the preparation of cryogels, the choice of the second monomer plays a decisive role in determining the final properties of the cryogels. Given that the previously prepared CG(AM) exhibited a relatively low epoxy-group density, three second monomers containing hydroxyl groups were selected with the aim of introducing hydroxyl functionalities into the cryogel system [43,44]. An increased presence of hydroxyl groups can provide more chemical modification sites, enabling subsequent epoxy activation and coupling to achieve efficient adsorption and removal of endotoxins.

Figure 3 presents high-resolution XPS spectra of CG(HEMA-co-AM), CG(SA-co-AM), and CG(PVA-co-AM), synthesized with HEMA, SA, and PVA as comonomers, respectively. In theregion (Figure 3b), Gaussian–Lorentzian deconvolution resolved three main components: a hydrocarbon C–C/C=C peak at 284.8 eV; a C–O/C–O–C peak at 286.2 eV, corresponding to ether and hydroxyl groups from the comonomers; and a carbonyl C=O (amide) peak at 288.1 eV, confirming acrylamide incorporation [45,46]. The spectra (Figure 3c) display two peaks at 531.6 eV (amide C=O oxygen) and 533.0 eV (C–O–H and C–O–C functionalities), while the region (Figure 3d) shows a single amide nitrogen peak at 399.6 eV with no detectable protonated amine signal near 401 eV [47]. A quantitative comparison of the deconvoluted peak areas indicates variations in the C=O and C–O ratios among the three cryogels, reflecting differences in comonomer content and corroborating the successful tailoring of surface chemistry via HEMA, SA, or PVA incorporation [48].

The FT-IR characterization results of the three cryogels (Figure 2a) show that free hydroxyl groups (hydroxyl groups not engaged in hydrogen bonding) exhibit absorption peaks in the high-wavenumber range (3650 cm^−1^ to 3580 cm^−1^), corresponding to the stretching vibrations of O-H bonds [42,49]. When hydroxyl groups form hydrogen bonds, whether intra- or intermolecular, the corresponding peaks shift to lower wavenumbers, appearing in the range of 3550 cm^−1^ to 3200 cm^−1^. Due to the complex three-dimensional network structure of cryogels, ample opportunities exist for hydrogen bond formation, making the hydrogen-bonding interactions within cryogels significantly more intricate than in simple molecules [50,51]. This complexity and the potential for multiple interactions may result in broadening or overlapping of peaks in the infrared spectra [52]. Taken together, the XPS and FT-IR characterization results confirm the successful preparation of all three cryogels.

The different second monomers, due to inherent differences in their physicochemical properties, significantly affect the performance of the cryogel systems. Although the amounts of second monomers added were kept constant, the copolymerization effects with the cryogel system—such as swelling degree, porosity, and the degree of rehydration after drying—are influenced by a complex interplay of factors, including, but not limited to, polymer compatibility, crosslinking density, hydrogen-bonding interactions with water molecules, and internal stresses generated during drying. The swelling degree represents the cryogel’s liquid absorption capacity and extent of expansion in a solvent, reflecting the looseness of its network structure and interactions with the solvent. Porosity indicates the volume fraction of internal voids within the cryogel, influencing mechanical properties, heat transfer, and adsorption performance [53].

As shown in Table 1, compared to CG(AM), CG(HEMA-co-AM) exhibited an 11.23% increase in average swelling degree, and the activated-epoxy-group density increased to 26.23 μmol/g, approximately a 17.57% improvement. When SA was used as the second monomer to form CG(SA-co-AM), the introduction of SA led to a highly viscous fluid state during cryogel preparation. This viscosity significantly affected the texture and consistency of the cryogel, resulting in shrinkage after drying and making it difficult to fully recover its original shape. This is because high-viscosity substances tend to form compact structures during dehydration, restricting the cryogel’s ability to revert to its initial form after drying [54]. Therefore, SA is not an ideal choice as a second monomer for cryogel preparation.

In contrast, when PVA was used as the second monomer to prepare CG(PVA-co-AM), both the swelling degree and porosity decreased relative to CG(AM), and no significant improvement in activated-epoxy-group density was observed, suggesting that PVA is limited in enhancing cryogel performance [55].

In conclusion, introducing HEMA as the second monomer significantly improved the swelling degree and epoxy-group density of the cryogels, thereby optimizing their overall performance. This can be attributed to HEMA’s excellent biocompatibility and hydrophilicity, making it a commonly used material in biomedical applications. Thus, HEMA is an ideal second monomer choice for enhancing cryogel properties.

**Figure 4:**
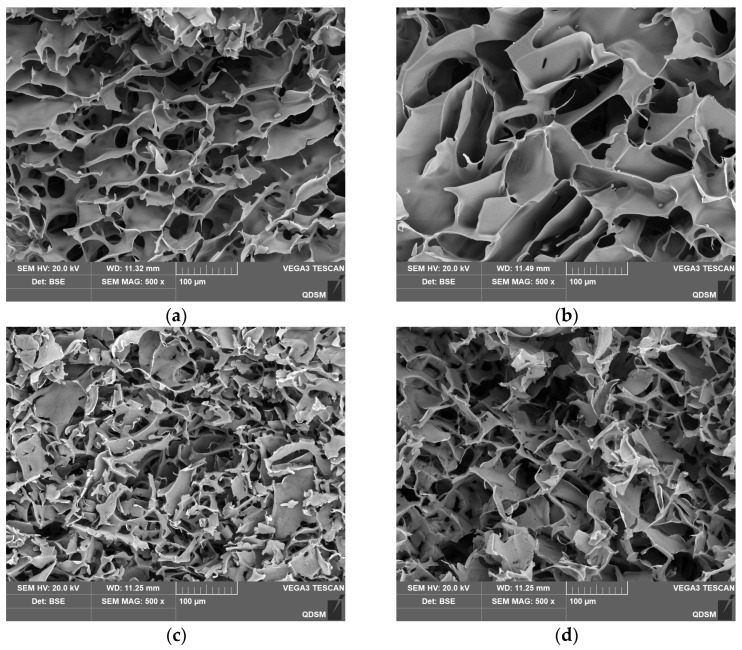
SEM characterization results of the cryogel: (**a**) CG(AM); (**b**) CG(HEMA-co-AM); (**c**) CG(SA-co-AM); (**d**) CG(PVA-co-AM).

The internal structure of the cryogels was further investigated using scanning electron microscopy (SEM), as shown in Figure 4. Compared to other cryogels, CG(HEMA-co-AM) exhibited a broader pore size distribution and a more ordered semi-interpenetrating network structure. This notable difference is primarily due to the high reactivity of the vinyl groups in HEMA, along with the ester and hydroxyl groups, which can form numerous crosslinking points and hydrogen bonds during polymerization, Moreover, phase separation during the polymerization process also significantly influenced the internal structure [56]. These combined factors endowed CG(HEMA-co-AM) with a wide pore size range (50–100 μm) and relatively thick pore walls (5–15 μm), with larger pore sizes than those reported for the poly(acrylic acid)/chitosan cryogels prepared by Zhang Min et al. (25 ± 7 μm) [57].

The large pore size facilitates diffusion and transport within the cryogel, while the thick pore walls provide enhanced mechanical support and stability, granting CG(HEMA-co-AM) significant advantages in chromatographic applications for solution sample processing.

### 2.3. Optimization of CG(HEMA-co-AM) Preparation

The effects of total polymer concentration on cryogel preparation are shown in Table 2. As the total polymer concentration increased, the activated-epoxy-group density of the cryogels also increased. However, both excessively high and low polymer concentrations led to decreases in swelling degree and porosity [58].

When the monomer concentration was less than or equal to 6%, the resulting cryogels were soft and structurally unstable, unable to meet subsequent processing requirements. In particular, when the monomer concentration was reduced to 5%, the cryogel structure could not form completely, indicating insufficient crosslinking to support cryogel formation at this concentration. When the concentration was increased to 8%, although cryogels could form, their excessive hardness negatively impacted subsequent epoxy activation, coupling, and endotoxin removal steps [59]. Thus, 7% was selected as the optimal total monomer concentration. At this concentration, the prepared CG(HEMA-co-AM) exhibited sufficient structural stability and moderate texture. The enhanced swelling degree reflected improved water absorption capacity, facilitating application in moist environments, while the optimized porosity increased the specific surface area and permeability, providing favorable conditions for subsequent experiments and real-sample applications.

By adjusting the monomer ratio, key properties such as the crosslinking density, pore size, and mechanical strength of the cryogels could be effectively controlled [60]. The effects of different monomer ratios on CG(HEMA-co-AM) are shown in Table 3. When the molar ratio of AM to HEMA was 2:1, the activated-epoxy-group density reached 30.45 μmol/g. Compared to other ratios, this ratio enabled the most effective copolymerization between AM and HEMA, forming a cryogel network rich in hydroxyl groups, thereby achieving structural stability along with appropriate flexibility. Under these conditions, the cryogels exhibited excellent an swelling degree, porosity, and activated-epoxy-group density.

### 2.4. Characterization Results of CG(HEMA-co-AM)

**Figure 5:**
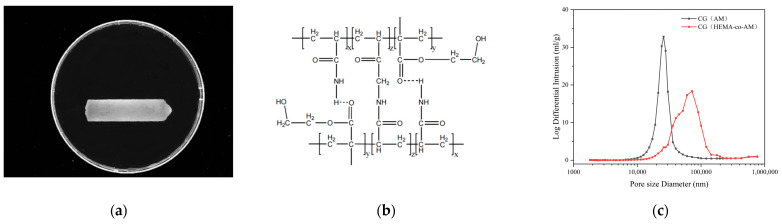
(**a**) Surface morphology of CG(HEMA-co-AM); (**b**) chemical structure of CG(HEMA-co-AM); (**c**) pore size distribution of CG(HEMA-co-AM).

By optimizing both the total polymer concentration and the monomer ratio during CG(HEMA-co-AM) synthesis, the cryogel’s performance was significantly enhanced. Under these optimal conditions, the surface morphology of CG(HEMA-co-AM) is shown in Figure 5a, and its structural features—deduced from FT-IR spectra and XPS analysis—are summarized in Figure 5b.

Mercury intrusion porosimetry exploits mercury’s non-wetting behavior and surface tension interactions with the material to precisely characterize pore architecture by measuring the volume of mercury intruded into the pores [61]. As plotted in Figure 5c, the macropores of CG(HEMA-co-AM) are concentrated predominantly in the 70–120 μm diameter range; by contrast, CG(AM) exhibits a narrower pore distribution of 25–45 μm.

The average pore diameter and median pore diameter of CG(HEMA-co-AM) are both substantially larger than those of CG(AM) (Table 4), and CG(HEMA-co-AM) even contains pores as large as 250 μm. These findings align with the SEM observations in Figure 4a,b. Relative to CG(AM), the optimized CG(HEMA-co-AM) demonstrates a 2.45-fold increase in average pore size. Such enlarged macropores enhance the cryogel’s capacity to accommodate large particles or biomolecules, thereby improving its efficacy in separation, purification, and adsorption applications [5,62]. Moreover, a macroporous network markedly boosts mass-transfer efficiency, enabling more rapid exchange and transport during processing [63,64].

**Figure 6:**
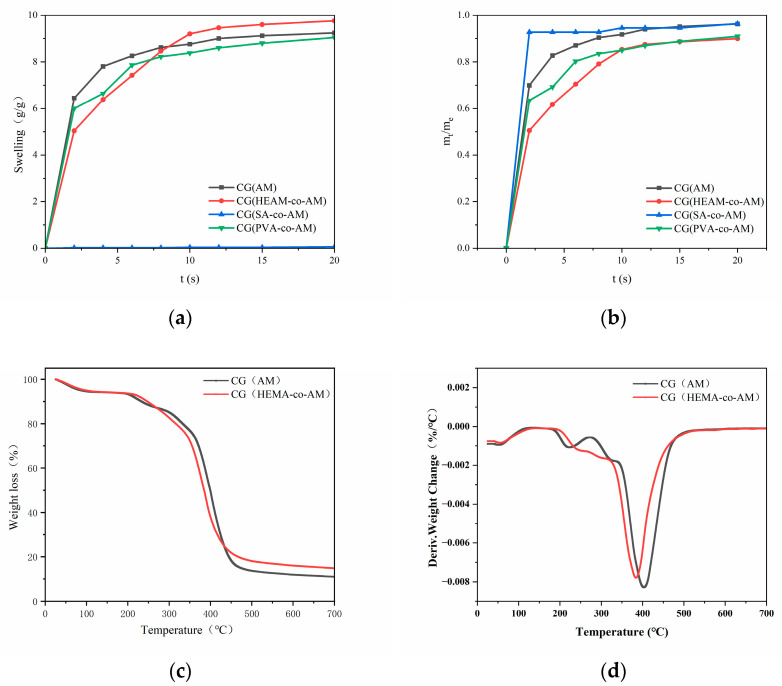
(**a**) Swelling curves of cryogels; (**b**) swelling kinetics of cryogels. (**c**) TGA thermogram of CG(HEMA-co-AM); (**d**) DTG curve of CG(HEMA-co-AM).

As shown in Figure 6a, except for CG(SA-co-AM)—whose collapsed pore structure renders it non-swelling—all other cryogels rapidly absorb water at first, then gradually slow until reaching equilibrium. Notably, CG(HEMA-co-AM) swells significantly more slowly than the other samples (Figure 6b), a phenomenon attributable to its larger pore architecture [65]. According to the Laplace–Kelvin equation, the driving force for liquid ingress is inversely proportional to pore radius [66]. Consequently, the larger pores in CG(HEMA-co-AM) yield a lower capillary driving force and hence a reduced initial swelling rate. However, at equilibrium, CG(HEMA-co-AM) attains the highest overall degree of swelling, indicating that its swelling behavior depends not only on pore size but also on its unique chemical composition and network structure [67].

After calculating and analyzing the swelling kinetic parameters (Table 5), the results indicate that the diffusional exponent (*n*) for CG(AM), CG(PVA-co-AM), and CG(HEMA-co-AM) is less than 0.5. This finding demonstrates that water diffusion in these cryogel systems predominantly follows Fickian diffusion [68]. The hallmark of Fickian diffusion is that the transport of water molecules is governed primarily by concentration gradients. Therefore, when interpreting the swelling behavior of these cryogels, the critical role of concentration gradients must be emphasized. Specifically, the magnitude of the concentration gradient not only dictates the rate of water diffusion within the cryogel matrix but also determines the direction of diffusion [69]. To investigate water diffusion within the cryogel during the swelling process, the swelling kinetics are described by the following equation:

(1) $$kt^{n}=\frac{m_{t}-m_{d}}{m_{e}-m_{d}}$$

where *k* is the characteristic constant of the cryogel, *t* is the time (s), and *n* is the characteristic exponent of the water diffusion model.

Given that CG(HEMA-co-AM) must undergo subsequent epoxy activation, ligand coupling, and endotoxin removal steps, it is essential to ensure that the cryogel possesses robust and stable physicochemical properties. The thermogravimetric analysis (TGA; Figure 6c) reveals that the primary mass losses for both cryogels occur between 220 °C and 430 °C. Below 100 °C, the observed weight loss is mainly attributed to the evaporation of residual moisture within the samples [70]. A minor weight-loss peak appears between 200 °C and 235 °C, likely corresponding to the degradation of epoxy functionalities and other labile groups [70]. A broader, more pronounced weight-loss event then occurs from 300 °C to 455 °C, corresponding to the decomposition of the cryogel backbone, as evidenced by a distinct plateau in the TGA curve. Comparing the thermal stability of CG(AM) and CG(HEMA-co-AM) shows that both the epoxy-group decomposition and backbone degradation in CG(AM) require higher temperatures to initiate. Importantly, CG(HEMA-co-AM) does not undergo complete thermal degradation below 300 °C, indicating that both cryogels exhibit excellent thermal stability, thus expanding their applicability in high-temperature environments.

**Figure 7:**
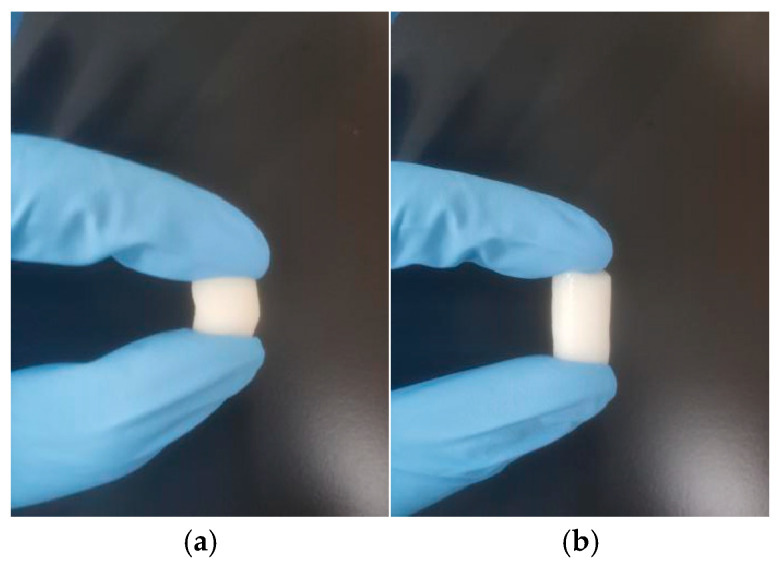
(**a**) Photograph of CG(HEMA-co-AM) compression; (**b**) photograph of CG(HEMA-co-AM) compression recovery.

As shown in Figure 7 and Table 6, all four cryogels exhibit superior compressive resilience compared to traditional particulate chromatographic media, which are prone to brittleness [71]. Notably, CG(HEMA-co-AM) remains crack-free under strains up to 70%, maintaining structural integrity and demonstrating the highest cohesion. Moreover, it recovers its original shape rapidly and completely upon stress release, showcasing exceptional elasticity (toughness and resilience). CG(SA-co-AM), in contrast, displays pronounced tackiness due to the viscous nature of its precursor solution [54]. Furthermore, CG(HEMA-co-AM) exhibits a maximum compressive stress of 18.8 kPa, surpassing both pre-freeze-fabricated gels and bulk PHEMA cryogel matrices and highlighting its exceptional mechanical performance [72,73].

### 2.5. Synthesis and Optimization of CG(HEMA-co-AM)@ECH

The epoxide activation efficiency of two common activators—1,4-butanediol diglycidyl ether (BDDE) and epichlorohydrin (ECH)—was compared (Table 7). Although their activation capacities are similar, BDDE’s longer spacer arm reduces steric hindrance and thus favors ligand coupling [74], it also makes the coupled PMB more prone to leaching due to excessive flexibility [75,76]. Balancing activation efficiency, ligand stability, and practical considerations, ECH was therefore selected for subsequent cryogel activation.

**Figure 8:**
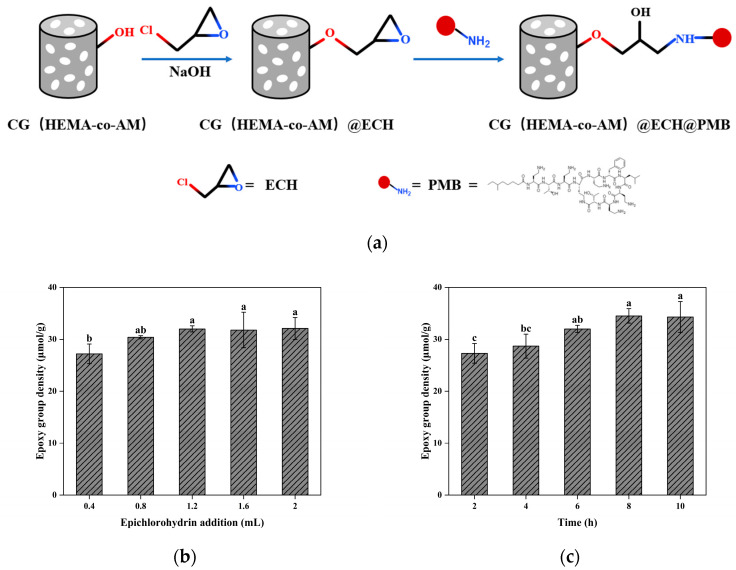
(**a**) Schematic diagram of the preparation of PMB-modified cryogel; (**b**) effect of epichlorohydrin addition on epoxy-group density; (**c**) effect of reaction time on epoxy-group density. Different letters (a, b, c) on the columns indicate significant difference between each other at *p* < 0.05 level.

As illustrated in Figure 8, CG(HEMA-co-AM) was first treated with ECH under alkaline conditions. ECH undergoes ring-opening with the cryogel’s surface hydroxyls to form ether linkages, thereby introducing additional epoxide groups. In a second step, PMB was grafted via nucleophilic attack of its amino groups on these epoxides, opening the epoxide rings and forming stable C-N bonds [38].

To optimize activation, we varied the ECH loading (0.4–2.0 mL) and reaction time (2–10 h). As shown in Figure 8c, the optimal condition—1.2 mL of ECH for 8 h—yielded a cryogel with swelling degree of 13.57, macroporosity of 76.64%, and epoxide density of 34.50 μmol/g, representing a 54.64% increase over unactivated CG(AM). This enhanced epoxide density provides more reactive sites for downstream functionalization.

### 2.6. Synthesis and Optimization of CG(HEMA-co-AM)@ECH@PMB

**Figure 9:**
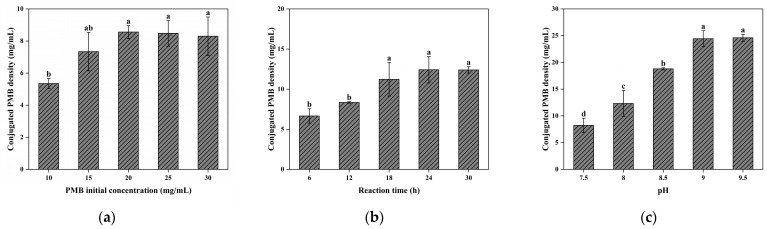
(**a**) Effect of initial PMB concentration on the coupling efficiency; (**b**) effect of reaction time on the coupling efficiency; (**c**) effect of pH on the coupling efficiency. Different letters (a, b, c, d) on the columns indicate significant difference between each other at *p* < 0.05 level.

The coupling of PMB to CG(HEMA-co-AM)@ECH relies on nucleophilic ring-opening of epoxides by PMB’s amino groups. This reaction proceeds slowly under neutral conditions and requires a strongly alkaline environment to accelerate. As shown in Figure 9c, raising the pH of the PMB coupling solution improved coupling efficiency. However, PMB solubility decreased under highly basic conditions, with visible precipitation occurring above pH 9.5 [77,78]. For balancing the PMB stability and coupling efficiency, pH 9 was identified as optimal, yielding a cryogel PMB density of 24.43 mg/mL.

### 2.7. Characterization of CG(HEMA-co-AM)@ECH@PMB

**Figure 10:**
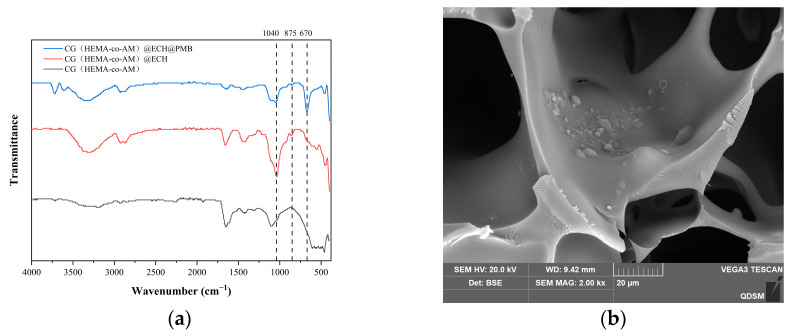
(**a**) FT-IR spectrum of the cryogel; (**b**) SEM characterization of CG(HEMA-co-AM)@ECH@PMB.

Comparison of FT-IR spectra before and after ECH activation (Figure 10a) reveals clear chemical changes. The broad band near 3434 cm^−1^ (O-H stretch) and the band at 910 cm^−1^ (epoxide ring vibration) confirm the presence of hydroxyl and epoxide groups in CG(HEMA-co-AM). Upon ECH activation, the peak at ~1040 cm^−1^—assigned to C-O-C stretching from newly formed ether bonds between cryogel hydroxyls and ECH—intensifies [79], and the epoxide vibration at ~875 cm^−1^ also increases [70], verifying successful epoxide grafting. After PMB conjugation, a new stretching vibration peak appearing at 670 cm^−1^ in the FTIR spectrum of CG(HEMA-co-AM)@ECH@PMB serves as strong evidence for the formation of C–N bonds [80]. According to the theory of chemical bond vibrations, this frequency falls within a reasonable range for C–N bond stretching vibrations and may exhibit shifts depending on the molecular structural environment. Referring to the relevant literature, similar coupling systems and structurally related compounds also display new IR peaks at comparable frequencies upon C–N bond formation [81]. Simultaneously, the XPS analysis confirms the presence of nitrogen (N) in CG(HEMA-co-AM)@ECH@PMB, with binding energy characteristics consistent with N atoms in C–N bonds [82,83]. SEM images (Figure 10b) further confirm the successful immobilization of PMB onto CG(HEMA-co-AM)@ECH, as evidenced by changes in surface morphology consistent with polymer coatings.

Together, these results validate the stepwise functionalization of the cryogel, culminating in CG(HEMA-co-AM)@ECH@PMB with dual electrostatic and hydrophobic binding sites for efficient endotoxin adsorption.

### 2.8. Evaluation of Endotoxin Adsorption by CG(HEMA-co-AM)@ECH@PMB

#### 2.8.1. Endotoxin Adsorption Performance in Tris-HCl Solution

**Figure 11:**
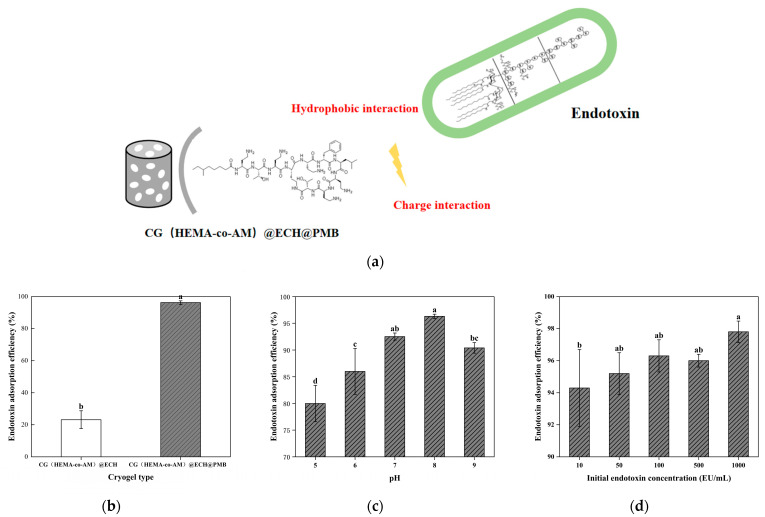
Adsorption performance of CG(HEMA-co-AM)@ECH@PMB cryogel: (**a**) mechanism of endotoxin adsorption; (**b**) influence of cryogel modification stages on endotoxin adsorption efficiency; (**c**) effect of pH on endotoxin adsorption capacity; (**d**) impact of initial endotoxin concentration on adsorption equilibrium. Different letters (a, b, c, d) on the columns indicate significant difference between each other at *p* < 0.05 level.

As illustrated in Figure 11, under physiological conditions the phosphate groups of lipid A carry a negative charge, whereas the PMB molecule bears five protonated amine groups (γ-aminobutyric acid residues) that are positively charged [77]. This electrostatic attraction drives the strong binding between PMB and endotoxin. In addition, PMB’s hydrophobic regions further enhance adsorption: its N-terminal 6-methyl-octanoyl chain interacts with the long-chain fatty acids of lipid A (e.g., B-OH myristic acid), reinforcing the overall binding affinity through hydrophobic interactions [78,84].

Brandenburg systematically investigated the thermodynamic parameters (ΔH, ΔS, ΔG) of PMB binding to LPS with different sugar chain lengths (including pure lipid A and various Re, Rc, Ra, and S-form LPS) in the temperature range of 20–50 °C using isothermal titration calorimetry (ITC). The results showed that the binding process was primarily driven by entropy-driven electrostatic interactions (ΔS remained positive), and hydrophobic interactions significantly enhanced the binding enthalpy (ΔH became more negative) with the increase in the acyl chain length of lipid A [85]. Schromm confirmed through zeta potential measurements that the surface charge rebounded from above −30 mV to approximately −5 mV after the complexation of PMB with endotoxin, revealing the mechanism of efficient neutralization of LPS negative charge by multivalent cationic sites of PMB [86]. Additionally, Wu designed PMB derivatives through molecular docking and identified that the positive charge density of diaminobutyric acid (DAB) residues was critical for determining the balance between endotoxin binding capacity and toxicity [87]. The constructed fluorescently labeled PMB derivatives exhibited a 25% increase in binding energy and possessed both antibacterial and imaging functions, providing a new strategy for targeted therapy. Specifically, the structural compatibility between polymyxin B and endotoxin originates from their molecular-level synergistic interaction mechanism: the positively charged DAB residues in the cyclic heptapeptide backbone of PMB form strong electrostatic interactions with the phosphate groups of LPS lipid A through protonated amino groups, while the fatty acid chain (capric acid) in its linear tail inserts into the hydrophobic core of the LPS lipid bilayer to enhance the binding stability via hydrophobic interactions [88,89]. Moreover, the topological flexibility of the cyclic structure enables dynamic adaptation to the conformational changes of lipid A, forming multi-site synergistic binding. Based on the above mechanisms, compared with traditional single-anion-exchange resins or linear cationic polymers, the cryogel materials designed in this study, such as CG(HEMA-co-AM)@ECH@PMB, not only retain the charge complementarity and hydrophobic anchoring properties through PMB immobilization, but their internal large-pore structure also matches the size of LPS aggregates, further amplifying the synergistic hydrophobic effect and achieving multivalent electrostatic–hydrophobic synergistic adsorption of endotoxin through a three-dimensional porous network.

To model adsorption in a controlled yet physiologically relevant system, we employed Tris-HCl buffer spiked with LPS and substituted complex biological fluids with defined protein solutions [90]. This approach minimizes sample-to-sample variability and cost while enabling systematic investigation of key parameters. As shown in Figure 11a, unmodified CG(HEMA-co-AM)@ECH removes only 28.7% of LPS under these conditions, reflecting non-specific adsorption by residual gel amino and hydroxyl groups [84]. In contrast, CG(HEMA-co-AM)@ECH@PMB achieves up to 96.2% endotoxin removal, confirming the effectiveness of PMB functionalization.

Figure 11b demonstrates that solution pH profoundly influences adsorption. At pH 5.0, removal falls below 80%, as protonation of LPS phosphate and carboxyl groups reduces their net negative charge and weakens electrostatic interactions with PMB [91,92]. Simultaneously, low pH alters the overall conformations of both LPS and PMB—polysaccharide and polypeptide molecules that adopt more compact structures at acidic pH—thereby modulating the distribution of hydrophobic versus hydrophilic surface domains [93,94]. Above pH 9.5, PMB begins to precipitate, limiting the coupling efficiency [77,78]. A compromise pH of ~8.0 yields optimal adsorption (>96%), where LPS and PMB achieve the ideal balance of ionization and conformational stability to maximize both electrostatic and hydrophobic binding.

Figure 11c examines the effect of the initial endotoxin concentration. CG(HEMA-co-AM)@ECH@PMB performs exceptionally well at high LPS loads, benefiting from its large-pore architecture that supports rapid percolation and minimal mass-transfer limitations. At low endotoxin levels, however, the fast flow rate can compromise contact time, reducing adsorption efficiency, and measurement error becomes more pronounced. These findings underscore the need to tailor experimental conditions—particularly flow rate and sample concentration—to the intended application to ensure reliable, reproducible endotoxin removal.

#### 2.8.2. Adsorption Kinetics

**Figure 12:**
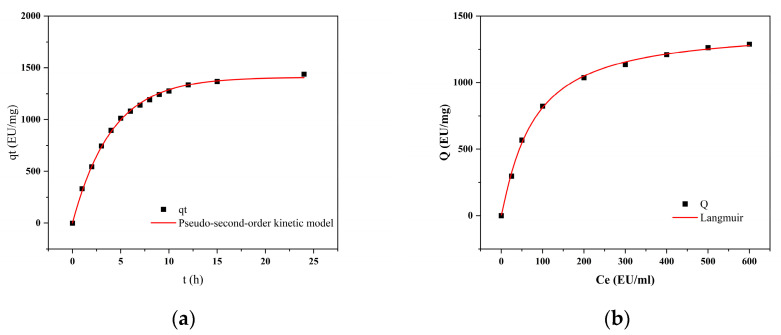
(**a**) The influence of adsorption time on the adsorption capacity; (**b**) adsorption isotherm.

The adsorption rate is a key parameter for purification efficiency. Figure 12a shows the amount of endotoxin adsorbed over time: it increases sharply from 0 to 40 min and reaches equilibrium at 60 min. The experimental data were linearly fitted to the pseudo-second-order kinetic model (Table 8), where q_e_ (EU/mg) represents the equilibrium adsorption capacity, q_t_ (mg/g) is the adsorption amount at time t (min), k (g·mg^−1^ min^−1^) is the pseudo-second-order rate constant, and DD is the correlation coefficient of the fit. The goodness of fit (R^2^ > 0.99) indicates that the adsorption process of CG(HEMA-co-AM)@ECH@PMB follows pseudo-second-order kinetics, implying that the overall rate is governed primarily by chemisorption [95].

Compared to similar systems reported in previous studies, the current material exhibits a faster adsorption rate and higher equilibrium adsorption capacity. For instance, Kavianpour reported a magnetic nanoparticle-based adsorbent requiring over 90 min to reach equilibrium [20], while in our system, saturation was achieved within 60 min. Moreover, the pseudo-second-order rate constant (k) and equilibrium capacity (qₑ) are both significantly higher than those reported for conventional polymer- or hydrogel-based LPS adsorbents [21,22]. These results highlight the superior endotoxin removal efficiency of CG(HEMA-co-AM)@ECH@PMB, likely due to the synergistic effect of the PMB moieties and the optimized porous network facilitating fast mass transfer.

#### 2.8.3. Adsorption Isotherm Experiments

As shown in Figure 12b, the amount of endotoxin adsorbed by CG(HEMA-co-AM)@ECH@PMB increases with the initial endotoxin concentration and plateaus at higher concentrations. Data were fitted to the classical Langmuir isotherm model, which assumes monolayer adsorption on a homogeneous surface with no interactions among adsorbed molecules [96,97]. In Table 8, C_eq_ denotes the equilibrium endotoxin concentration, Q the adsorption capacity at equilibrium, K_d_ the dissociation constant, and Q_max_ the theoretical maximum adsorption capacity. This excellent fit demonstrates that the adsorption process of endotoxin onto this material aligns closely with the Langmuir model. The theoretical maximum adsorption capacity Q_max_ derived from the Langmuir model is 1408.38 EU/mg, which far exceeds the endotoxin adsorption performance of certain traditional materials such as resins and nanoparticles [98,99]. This provides a critical benchmark for evaluating the cryogel’s potential in the field of endotoxin removal. The strong agreement with the Langmuir model indicates that adsorption on CG(HEMA-co-AM)@ECH@PMB approaches ideal monolayer behavior, with uniform surface properties and minimal adsorbate–adsorbate interactions [100].

#### 2.8.4. Endotoxin Adsorption Performance in Protein Solutions

**Figure 13:**
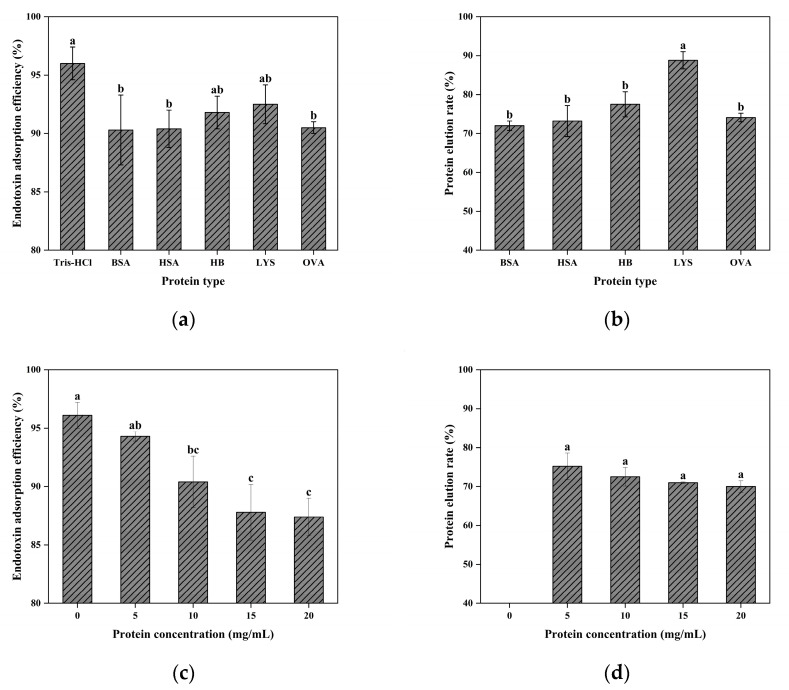
(**a**) The effect of sample type on the adsorption of endotoxin; (**b**) effect of sample type on protein recovery. (**c**) The influence of protein concentration on the adsorption of endotoxin; (**d**) effect of protein concentration on protein recovery. Different letters (a, b, c) on the columns indicate significant difference between each other at *p* < 0.05 level.

The macroporous architecture of the cryogel enhances mass-transfer efficiency in complex sample matrices but can reduce the surface contact area and affinity-ligand density, potentially compromising adsorption performance [64]. Although CG(HEMA-co-AM)@ECH@PMB achieved over 96.05% endotoxin removal in Tris-HCl buffer and maintained ≥90.30% removal in various protein-containing solutions (Figure 13a), these results slightly underperform commercial columns rated at 99.90% efficiency [101], indicating that the large-pore structure has inherent limitations. Optimization of the flow rate and other operational parameters may further improve performance.

Figure 13b shows that lysozyme (LYS) exhibited the highest protein recovery (88.82%) after a single pass, while hemoglobin (Hb) achieved 77.56%, outperforming other proteins. In contrast, bovine serum albumin (BSA), human serum albumin (HSA), and ovalbumin (OVA) showed the lowest recoveries, with BSA at only 72%. Mechanistic investigations suggest that the dominant interaction responsible for binding endotoxins and proteins is electrostatic in nature. Under acidic conditions, the positively charged PMB ligands attract negatively charged biomolecules through Coulombic interactions [77]. Hydrophobic interactions also contribute, especially for moderately hydrophobic proteins (BSA, HSA, OVA), exacerbating their non-specific loss. These proteins (pI 4.5–4.7) carry significant negative charge and moderate hydrophobicity at pH 8.0, matching both the cationic and hydrophobic domains of PMB, thus promoting non-specific adsorption [101,102]. LYS (pI ≈ 11) is positively charged and weakly hydrophobic at pH 8.0, resulting in minimal non-specific adsorption and the highest elution yield. Hb, with near-neutral pI and strong hydrophobicity, displays intermediate adsorption-recovery behavior.

As shown in Figure 13, increasing the protein concentration leads to slight decreases in endotoxin removal and protein elution. High protein levels promote endotoxin–protein complex formation, altering surface properties and creating steric hindrance within gel pores, thereby inhibiting binding to PMB sites. Competitive non-specific binding of proteins to PMB sites may further reduce adsorption efficiency.

In summary, under gravity-driven flow, CG(HEMA-co-AM)@ECH@PMB achieves >90% endotoxin removal in protein solutions, with selectivity and protein recovery strongly influenced by target molecules’ pI and hydrophobicity. Future enhancements could include tailoring the pore size, raising the affinity-ligand density, and adjusting the ionic strength to optimize the adsorbent design for diverse biomolecules.

#### 2.8.5. Effect of Flow Rate on Endotoxin Adsorption and Protein Recovery

**Figure 14:**
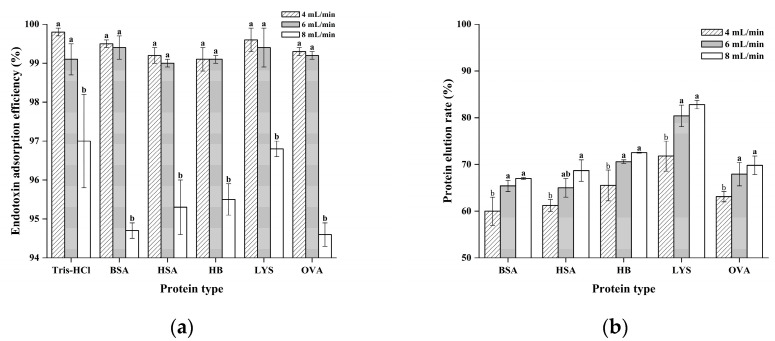
(**a**) The influence of perfusion rate on the adsorption of endotoxin; (**b**) influence of perfusion rate on protein recovery. Different letters (a, b) on the columns indicate significant difference between each other at *p* < 0.05 level.

The flow rate critically affects both endotoxin adsorption and protein elution. As illustrated in Figure 14, slowing the flow from 8 to 4 mL/min increases endotoxin removal by CG(HEMA-co-AM)@ECH@PMB from 94.70% to 99.52% in BSA solutions but decreases BSA recovery from 67.12% to 60.32%. Reduced flow prolongs contact time between endotoxin and PMB ligands, enhancing adsorption; however, extended exposure also increases non-specific protein adsorption and steric hindrance within the gel, thus lowering protein elution [103,104]. Moreover, slower flow may favor endotoxin–protein complex formation, further complicating adsorption and quantification.

In previous studies, traditional resin-based materials have generally faced bottlenecks of limited endotoxin removal efficiency and significant non-specific adsorption [98,105]. The endotoxin removal efficacy of nanomaterials is highly dependent on surface functionalization properties and is significantly disturbed by competitive protein adsorption in complex biological systems. For example, the core–shell nanoparticles developed by Puja adsorb endotoxins through hydrophobic interactions of C18 chains, but their elution process relies on the introduction of surfactants [106]. This not only may disrupt the structural compatibility of proteins but also interfere with chromogenic assays for endotoxin detection, necessitating additional post-processing steps for surfactant removal. Balancing endotoxin removal and protein recovery, a flow rate of 6 mL/min was selected as optimal for CG(HEMA-co-AM)@ECH@PMB. Under these conditions in Tris-HCl buffer, endotoxin removal reached 99.82%, and up to 99.62% in protein solutions—avoiding excessive non-specific adsorption. Compared to conventional chromatography, this approach delivers a 4-fold increase in flow rate over standard columns, an 8-fold improvement over commercial adsorbents [101,107], and consistently >99% endotoxin removal, all without any observed clogging, demonstrating excellent permeability and operational stability.

#### 2.8.6. Effect of Reuse on Endotoxin Adsorption and Protein Recovery

**Figure 15:**
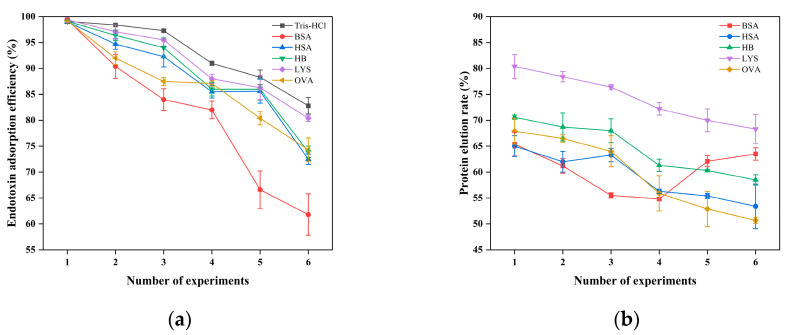
(**a**) The effect of the number of experiments on the adsorption of endotoxin; (**b**) influence of experimental replicate number on protein recovery.

Figure 15 shows that as the number of use cycles of CG(HEMA-co-AM)@ECH@PMB increases, the endotoxin removal efficiency of all samples declines to varying degrees, and most samples exhibit a gradual decrease in protein recovery. Several factors likely contribute. First, with each cycle, adsorbed endotoxin accumulates within the cryogel and occupies a fraction of the available binding sites. Second, non-specific adsorption of proteins and formation of protein–endotoxin complexes further occupy pore volume and narrow transport channels. Although no severe clogging was observed, the build-up of these complexes nonetheless impairs both endotoxin capture and protein elution. Importantly, the cryogel’s macroporous architecture preserves adequate flow performance throughout repeated use, without the pressure-induced flow obstruction or seal failure reported for some conventional endotoxin adsorbents [108,109,110].

Despite CG(HEMA-co-AM)’s demonstrated physicochemical stability in preliminary characterization, perfusion inevitably imposes mechanical and chemical stresses that can damage its internal structure over multiple cycles [111]. In particular, regions near the gel core—where slower heat transfer during synthesis may have left incomplete polymerization—tend to exhibit localized shrinkage and heterogeneous porosity upon freeze-drying [33]. Under repeated perfusion, these structural weaknesses become pronounced, leading to internal collapse and a concomitant loss of adsorption sites, which in turn reduces both endotoxin removal and protein recovery. For example, in the BSA series, endotoxin removal dropped precipitously after the fifth cycle and fell to just 61.8% by the sixth, while protein recovery spiked in the fifth cycle (Figure 15b). This behavior likely reflects partial structural failure that allows protein to elute without sufficient endotoxin clearance [112]. Future studies should explore material redesign or process modifications to slow performance degradation, extend operational lifetime, and maintain high adsorption efficiency.

#### 2.8.7. Effect of Ionic Strength on Endotoxin Adsorption and Protein Recovery

**Figure 16:**
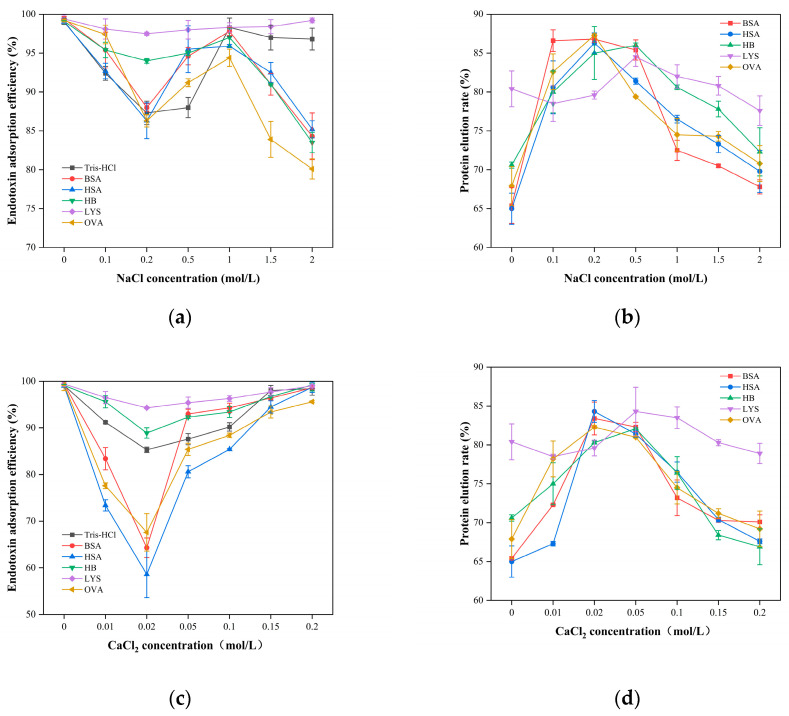
(**a**) The influence of NaCl concentration on the adsorption of endotoxin; (**b**) influence of NaCl concentration on protein recovery; (**c**) the effect of CaCl_2_ concentration on the adsorption of endotoxin; (**d**) influence of CaCl_2_ concentration on protein recovery.

Figure 16a illustrates that in a Tris-HCl system containing only endotoxin, increasing the NaCl concentration from 0 to 2 mol/L produces a non-monotonic adsorption profile: initial increase, subsequent decrease, and secondary rise. The effect is most pronounced for OVA, whose adsorption falls by 19.1% at 2 mol/L NaCl. Similarly, raising CaCl_2_ from 0 to 0.2 mol/L yields an initial drop in adsorption followed by recovery (Figure 16c), reflecting the competing influences of electrostatic screening and enhanced hydrophobic interactions as the ionic strength varies.

At low ionic strength (NaCl 0–0.2 M; CaCl_2_ 0–0.02 M), endotoxin adsorption is dominated by electrostatic attraction between its anionic phosphate groups and the cationic PMB ligands. Initial increases in salt concentration strengthen ionic screening more than they enhance hydrophobic interactions, resulting in partial endotoxin desorption and reduced adsorption. In the moderate ionic strength range (NaCl 0.2–1.0 M; CaCl_2_ 0.02–0.2 M), disruption of the hydration shell around both endotoxin and PMB exposes hydrophobic domains, and hydrophobic driving forces surpass electrostatic weakening, restoring adsorption. At high ionic strength (NaCl 1.0–2.0 M), excess mono- and divalent ions compete directly for PMB binding sites and form a Debye screening layer that neutralizes surface charges and induces endotoxin aggregation, causing adsorption efficiency to decline once more [113].

A breakthrough-curve analysis for five proteins (BSA, HSA, Hb, OVA, LYS) shows that, except for LYS, protein elution exhibits a “rise-then-fall” trend with increasing salt (Figure 16b,d). At low-to-moderate salt, screening reduces electrostatic binding to PMB, increasing elution; at higher salt, stronger hydrophobic interactions lead to partial re-adsorption and a slight drop in elution. LYS, being positively charged and weakly hydrophobic at pH 8.0, experiences minimal electrostatic attraction or hydrophobic binding to PMB, maintaining > 90% recovery and insensitivity to ionic strength changes [114].

For BSA (pI ≈ 4.7, moderate hydrophobicity), increasing NaCl from 0 to 1.0 M in pH 8.0 Tris-HCl weakens its electrostatic interaction with PMB, raising elution from 35% to 80% [114]. Further increasing NaCl to 1.0–2.0 M strengthens hydrophobic binding enough to overcome electrostatic loss, reducing elution to 70% [115].

Ca^2^⁺ exerts more complex effects on the BSA–PMB–endotoxin system. In 0–0.02 M CaCl_2_, Ca^2^⁺ both screens electrostatics and bridges BSA–endotoxin complexes, lowering endotoxin removal to 64% while BSA recovery rises to 85% [116]. Without CaCl_2_, BSA retains a strong negative charge, yielding only 65.2% elution; with CaCl_2_, ongoing screening and weak BSA hydrophobicity result in continued elution increase. HSA and OVA, with similar charge and hydrophobicity, follow the same trend.

In hemoglobin (Hb, pI near neutral, strong hydrophobicity), Ca^2+^ bridges Hb–endotoxin complexes and reduces removal (Figure 16a,c). Under equal ionic strengths (CaCl_2_:NaCl at a 2:5 ionic strength ratio), both salts similarly affect endotoxin removal, indicating that ion identity significantly influences adsorption behavior. At 0.2 mol/L CaCl_2_ (equivalent to 0.5 mol/L NaCl ionic strength), Hb elution drops sharply due to Ca^2^⁺-enhanced hydrophobic interactions with the gel matrix [117].

Lysozyme (LYS, pI ≈ 11, weakly hydrophobic) undergoes minimal non-specific adsorption on CG(HEMA-co-AM)@ECH@PMB at pH 8.0 and maintains high recovery across salt concentrations, owing to uniform charge distribution, fewer endotoxin-binding sites, and high solubility that resists salt-induced precipitation or adsorption [25,118,119].

In summary, increasing ionic strength both enhances hydrophobic interactions and attenuates electrostatic forces, producing a triphasic adsorption response: electrostatic dominance at low salt; hydrophobic dominance at moderate salt; and overall suppression at high salt due to screening and ion competition. The cooperative interplay of these two mechanisms underlies effective the endotoxin capture by PMB-modified cryogels. In practical applications, researchers should select an appropriate ionic strength based on the charge and hydrophobic characteristics of the sample, as this is crucial for achieving optimal endotoxin removal efficiency.

## 3. Conclusions

In this study, a functionalized macroporous cryogel, CG(HEMA-co-AM)@ECH@PMB, was successfully developed to address the limitations of conventional endotoxin adsorbents in handling high-concentration samples, particularly the low endotoxin removal efficiency and poor protein recovery. The cryogel was fabricated via a cryopolymerization strategy using hydroxyethyl methacrylate as a second monomer and acrylamide as the primary monomer, forming a porous network with interconnected channels (70–120 μm) and thick pore walls (5–15 μm). The material exhibited excellent swelling behavior, stable physicochemical properties, and outstanding mechanical resilience, with a maximum compressive stress of 18.8 kPa at 70% strain and complete recovery after repeated cycles. Upon epoxy activation and PMB coupling, the epoxy-group density increased by 54.6%, and the PMB loading reached 24.4 mg/mL. Under flow perfusion conditions (6 mL/min in Tris-HCl buffer), the cryogel achieved a maximum endotoxin removal rate of 99.82% and an adsorption capacity of 1408.38 EU/mg. In complex protein matrices containing BSA, HSA, Hb, LYS, and OVA (each 10 mg/mL), the cryogel retained over 90% endotoxin removal efficiency in a single cycle, with protein recoveries exceeding 72%—notably 88.8% for LYS—demonstrating excellent biocompatibility and selectivity. Further investigations revealed that pH and ionic strength played crucial roles in modulating the cryogel’s hydrophobic and electrostatic interactions with endotoxin. Owing to its facile preparation, scalability, high adsorption capacity, low non-specific binding, and superior dynamic separation performance, this reusable cryogel offers a promising solution for efficient endotoxin clearance in recombinant protein purification and clinical blood purification, while also providing new insights into the design of dynamic chromatographic media.

## 4. Materials and Methods

### 4.1. Materials

Acrylamide (AM, 98%, Sigma-Aldrich, St. Louis, MO, USA), sodium alginate (SA, pharmaceutical grade, Mw ≈ 2 × 10^5^ g/mol, Macklin Biochemical, Shanghai, China), poly(vinyl alcohol) (PVA, Mw 27,000 g/mol, hydrolysis degree 98–99%, Macklin Biochemical, Shanghai, China), N,N′-methylenebisacrylamide (MBAm, 99%, Sigma-Aldrich, St. Louis, MO, USA), epichlorohydrin (ECH, 99%, Sinopharm, Beijing, China), N,N,N′,N′-tetramethylethylenediamine (TEMED, 99%, Sigma-Aldrich, St. Louis, MO, USA), ammonium persulfate (APS, 98%, Sigma-Aldrich, St. Louis, MO, USA), allyl glycidyl ether (AGE, 97%, Sigma-Aldrich, St. Louis, MO, USA), 2-hydroxyethyl methacrylate (HEMA, 99%, Macklin Biochemical, Shanghai, China), tris(hydroxymethyl)aminomethane (Tris, 99%, Sigma-Aldrich, St. Louis, MO, USA), 1,4-butanediol diglycidyl ether (BDDE, 95%, Sinopharm, Beijing, China), dimethyl sulfoxide (DMSO, 99.9%, Sinopharm, Beijing, China), ammonia solution (NH₃·H_2_O, 25–28%), acetone (99.5%), glucose (99.5%), polymyxin B (PMB, 95% and 10,000 IU/mg activity, Solarbio, Beijing, China), phenol (99%, Solarbio, Beijing, China), polyethylene glycol 6000 (PEG6000, 99%, Solarbio, Beijing, China), DNase I (1000 U/mg, Solarbio, Beijing, China), RNase I (100 U/mg, Solarbio, Beijing, China), Proteinase K (30 U/mg, Tiangen, Beijing, China), potassium bromide (KBr, 99%, Macklin Biochemical, Shanghai, China), human serum albumin (HSA, 96%, Solarbio, Beijing, China), hemoglobin (Hb, 95% heme content 90%, Solarbio, Beijing, China), lysozyme (LYS, 90%, Solarbio, Beijing, China), ovalbumin (OVA, 98%, Solarbio, Beijing, China), and bovine serum albumin (BSA, 98%, Solarbio, Beijing, China) were used as received. Deionized water was employed throughout.

### 4.2. Preparation of CG(HEMA-co-AM)@ECH@PMB

#### 4.2.1. Synthesis of CG(AM)

The preparation of acrylamide cryogel CG(AM) was carried out according to previously reported reaction systems with optimization [62,64]. Precisely 210 mg of AM and 56 mg of MBAm, together with 35 μL of AGE, were dissolved in 4.5 mL of deionized water to form precursor solution A. To ensure purity, solution A was subjected to ice-bath stirring and vacuum degassing. Separately, 6 mg of APS was dissolved in 0.5 mL of sonicated deionized water to yield solution B. Under ice-bath conditions, 7.5 μL of TEMED was added to solution A, which was then vacuum-stirred at 300 rpm for 15 min to remove dissolved gases. Solutions A and B were mixed thoroughly and degassed again under ice-bath conditions for 1.5 min. The resulting mixture was rapidly transferred into a precooled, sealed 5 mL syringe (inner diameter = 13 mm) and polymerized at −13 °C for 24 h in a constant-temperature bath. After polymerization, the cryogel was thawed at room temperature, washed repeatedly with deionized water, then pre-frozen at −80 °C and freeze-dried. The final product was designated CG(AM).

#### 4.2.2. Selection of Second Monomer

To investigate the influence of the second monomer type on the cryogel properties, three distinct precursor solutions were prepared following a univariate approach. Each solution contained 105 mg of a secondary monomer (HEMA, SA, or PVA) mixed with AM at a 1:1 mass ratio, while maintaining constant amounts of crosslinker (MBAm) and coupling agent (AGE). After degassing in an ice bath, APS initiator solution was introduced, and the mixture was immediately injected into precooled syringes. Polymerization was carried out at −13 °C for 24 h. The resultant cryogels were thawed, washed with deionized water, lyophilized, and designated as CG(HEMA-co-AM), CG(SA-co-AM), and CG(PVA-co-AM), respectively.

#### 4.2.3. Optimization of CG(HEMA-co-AM) Preparation Conditions

In accordance with the procedure described in Section 4.2.1, the total polymer concentration (*w*/*v*) of AM, HEMA, MBAm, and AGE was systematically investigated at 5%, 6%, 7%, 8%, and 9% (*w*/*v*), with all other synthesis parameters held constant. This sequential variation allowed the isolation of concentration-dependent effects without multifactorial interference. Following optimization of the total polymer concentration, the molar ratio of monomers (AM∶HEMA) was evaluated as the subsequent variable. With the total polymer concentration fixed at the optimized 7% (*w*/*v*), molar ratios of 1:3, 1:2, 1:1, 2:1, and 3:1 were systematically tested under identical reaction conditions.

#### 4.2.4. Optimization of Epoxy-Activation Conditions

Two common epoxy-activation agents were evaluated. First, 0.5 g of CG(HEMA-co-AM) was washed repeatedly with deionized water to remove surface impurities and unbound ions, then treated with 2 mol/L NaOH to ensure an alkaline environment. The cryogel was immersed in 30 mL of NaOH solution (pH 8.0), and 0.8 mL each of ECH and BDDE were added. DMSO was introduced dropwise to enhance miscibility. The reaction proceeded at 37 °C and 180 rpm for 6 h.

To systematically evaluate the effect of each variable on the synthesis, the influence of ECH volume was first investigated by fixing the reaction conditions at 37 °C, 180 rpm, and a constant reaction time. ECH volumes of 0.4, 0.8, 1.2, 1.6, and 2.0 mL were individually applied to the reaction system. Following activation, the cryogels were capped by treatment with 1 mol/L glycine solution (pH 8.0) to block unreacted hydroxyl groups, and the resultant product was designated as CG(HEMA-co-AM)@ECH. Subsequently, the reaction time dependency was explored under optimized ECH volume conditions. Reaction durations of 2, 4, 6, 8, and 10 h were systematically tested at 37 °C and 180 rpm, with all other parameters held constant. After each reaction, the same post-treatment procedure using glycine solution was applied to terminate non-specific reactions and stabilize the cryogel structure.

#### 4.2.5. Optimization of PMB Coupling Conditions

Five milliliters of CG(HEMA-co-AM)@ECH was added to 30 mL of PMB solution and reacted at 37 °C and 120 rpm. The mixture was then transferred into 30 mL of 1 mol/L ethanolamine (pH 9.0) and reacted under the same conditions for 4 h to quench the remaining active groups. A 5% NaBH₄ solution was subsequently applied to reduce and stabilize any unsaturated structures. The final cryogel was extensively washed with deionized water, stored in 20% ethanol at 4 °C, and designated CG(HEMA-co-AM)@ECH@PMB.

Under otherwise constant conditions, the effects of individual parameters on the coupling efficiency of polymyxin B (PMB) to the cryogel matrix were systematically investigated through a series of stepwise experiments. In each experiment, a single variable was adjusted while all other conditions were kept constant. First, the pH of the reaction buffer was varied across 7.5, 8.0, 8.5, 9.0, and 9.5 to evaluate the influence of pH on the coupling reaction. Subsequently, the reaction time was adjusted to 6, 12, 18, 24, and 30 h to assess how prolonged incubation affects coupling efficiency. Finally, the initial PMB concentration was varied between 10, 15, 20, 25, and 30 mg/mL to determine the effect of reagent dosage on the extent of immobilization. After each reaction, the amount of PMB immobilized on the cryogel was quantified using liquid-phase mass spectrometry. The PMB loading density was calculated based on the difference between the initial and residual PMB concentrations in the reaction solution.

#### 4.2.6. Determination of Epoxy-Group Density

Following the methods of previous studies, the epoxy-group density of the cryogel was determined using the sodium thiosulfate titration method [42,49,120]. One gram of freeze-dried cryogel was placed in a 25 mL conical flask. Ten milliliters of 1.3 mol/L sodium thiosulfate solution containing phenolphthalein indicator was added, and the mixture was incubated at 37 °C for 30 min with continuous mixing. The residual thiosulfate was titrated with standardized 0.1 mol/L HCl. The endpoint was reached when the solution turned from red to colorless and remained stable for over 30 s. The volume of HCl consumed was recorded for the epoxy density calculation:

(2) $$S=\frac{M_{HCl}\times \left(V_{0}-V_{1}\right)}{m}\times 100\%$$

where S is the epoxy density (mol/L), M_HCl_ is the concentration of HCl used (mol/L), V_0_ and V_1_ are the volumes of HCl before and after titration (mL), respectively, and m is the mass of the cryogel (g).

#### 4.2.7. Determination of PMB Density on the Cryogel

The PMB coupling solution remaining after reaction with the cryogel was analyzed by liquid chromatography–mass spectrometry (LC-MS) to determine the concentration of residual PMB, from which the density of PMB conjugated to the cryogel was calculated.

Chromatographic conditions: An Agilent HSS T3 column (2.1 × 100 mm, 1.8 μm) was used. The mobile phase consisted of 80% aqueous 0.1% formic acid (solvent A) and 20% acetonitrile containing 0.1% formic acid (solvent B), operated under isocratic elution. The column temperature was 30 °C, flow rate was 0.3 mL/min, and injection volume was 5 μL.

Mass spectrometry conditions: Electrospray ionization in positive mode (ESI^+^) was employed with multiple reaction monitoring (MRM). The capillary voltage was set to 4000 V, drying gas flow to 8 mL/min at 350 °C, and nebulizer pressure to 30 psi. The qualifier and quantifier ion pairs, along with collision energies, are listed in Table 9.

### 4.3. Evaluation of Endotoxin Adsorption Performance

#### 4.3.1. Gravity-Driven Adsorption

Tris-HCl buffer (100 EU/mL LPS, pH 8.0) served as the model sample. A mass of 0.5 g of cryogel was packed into a syringe and rinsed thoroughly with PBS (pH 7.4) to ensure uniform distribution and absence of air bubbles. The syringe outlet was connected to a 50 mL sample reservoir, and the sample was allowed to flow through the cryogel under gravity. After complete elution, three bed volumes of PBS were used to wash the gel; the wash fractions were collected for analysis. One complete adsorption experiment comprised three sequential cycles of sample loading and wash. The endotoxin removal efficiency was assessed by comparing inlet and eluent LPS concentrations.

After three loading-wash cycles, the cryogel was eluted with 2 mol/L NaCl to desorb bound endotoxin; the eluates were collected for quantitative analysis. Non-specific contaminants were removed by sequential elution with 0.2 mol/L NaCl and then 20 mmol/L PBS containing 1% sodium deoxycholate (pH 7.4). The gel was finally rinsed with pyrogen-free water to remove residual proteins and other impurities. All the eluates were retained. The cryogel was equilibrated in PBS (pH 7.4) until drainage ceased, then stored in 20% ethanol at 4 °C for future use.

#### 4.3.2. Adsorption Kinetics and Isotherm Experiments

Adsorption kinetics and isotherm experiments were carried out following the method of Wang and Su [121,122], with slight modifications. Precisely weigh 0.10 g of dried cryogel into a conical flask and add 50 mL of endotoxin solution (5000 EU/mL), ensuring the gel is fully submerged. Place the flask in a 37 °C water bath without agitation to simulate physiological adsorption. At predetermined time points (0, 4, 8, 12, 16, 20, 24, 28, 32, 36, 40, 50, 60, and 100 min), withdraw aliquots to determine residual endotoxin concentration. Calculate the amount adsorbed at each time point and plot the adsorption kinetics curves.

For isotherm studies, accurately weigh 0.10 g of dried cryogel into separate flasks, each containing 50 mL of endotoxin solutions at initial concentrations of 0, 33, 66, 100, 200, 300, 400, 500, and 600 EU/mL. Ensure the gel is fully immersed, then incubate all samples at 37 °C with gentle shaking for 100 min. After incubation, measure the final endotoxin concentrations, calculate equilibrium adsorption amounts, and construct adsorption isotherms. Fit the data to appropriate models to elucidate adsorption mechanisms and characteristics.

#### 4.3.3. Effect of Sample Parameters on Endotoxin Adsorption and Protein Breakthrough

Endotoxin adsorption and protein elution assays were conducted based on the protocols of Agustin and Reay [123,124], with appropriate modifications to enhance performance. To investigate the factors influencing the endotoxin adsorption performance of the cryogel, a series of experiments were carried out in a stepwise manner based on the procedure described in Section 4.3.1. In each set of experiments, a single variable was altered while keeping all other conditions constant to evaluate its individual effect.

First, the pH of the Tris-HCl buffer was adjusted to 5.0, 6.0, 7.0, 8.0, and 9.0 to examine the impact of environmental pH on adsorption efficiency. Subsequently, the initial endotoxin concentration was varied across 10, 50, 100, 500, and 1000 EU/mL to assess the influence of endotoxin loading on the adsorption behavior and capacity of the cryogel matrix. Next, five different protein types—bovine serum albumin (BSA), human serum albumin (HSA), hemoglobin (Hb), lysozyme (LYS), and ovalbumin (OVA)—were tested individually at a fixed concentration of 10 mg/mL to explore the selectivity of the adsorbent toward endotoxins in the presence of proteins with varying molecular weights and isoelectric points. Finally, BSA was used as a model protein, and its concentration was adjusted to 0, 5, 10, 15, and 20 mg/mL to investigate the effect of increasing protein load on endotoxin adsorption under competitive conditions.

For each experimental condition, the effluent was collected and analyzed to determine endotoxin adsorption efficiency, adsorption capacity, selectivity, protein breakthrough, endotoxin removal rate, and protein retention. All experiments were conducted in triplicate, and the results were averaged for analysis.

#### 4.3.4. Effect of Flow Rate on Endotoxin Adsorption and Protein Breakthrough

Following Section 4.3.1 and following Yue’s method [125], connect the bottom outlet of the syringe column to a peristaltic pump and adjust the flow rate to control sample perfusion through the cryogel. After three loading–wash cycles, switch the pump to deliver eluent through the gel to desorb bound endotoxin, and collect all eluates for analysis.

Investigate the impact of perfusion flow rates (4, 6, 8 mL/min), number of repeats (1–6 cycles), and ionic strength (final NaCl 0, 0.1, 0.2, 0.5, 1.0, 1.5, 2.0 mol/L; CaCl2 0, 0.01, 0.02, 0.05, 0.1, 0.15, 0.2 mol/L). Measure endotoxin and protein concentrations in the effluent. In a 20 mmol/L Tris-HCl buffer (pH 8.0) containing 500 EU/mL endotoxin and 10 mg/mL of each protein (BSA, HSA, Hb, LYS, OVA), assess the cryogel’s endotoxin adsorption performance and protein breakthrough behavior of CG(HEMA-co-AM)@ECH@PMB.

### 4.4. Cryogel Characterization

FT-IR analysis: Grind the fully dried cryogel into a fine powder. Subsequently, weigh 1–2 mg of the sample powder and thoroughly mix it with 100–200 mg of spectroscopic-grade KBr in an agate mortar by grinding for more than 10 min. Transfer the resulting mixture into a pellet die and compress it into a transparent thin disc under a pressure of 10–15 MPa for 5–10 min. Prior to measurement, calibrate the FT-IR spectrometer using a polystyrene film. The spectral resolution should be set to 4 cm^−1^, with 32 scans per measurement. Place the pressed pellet into the sample holder, ensuring the light path is fully covered, and perform the scan. The collected data are subjected to baseline correction and smoothing. Characteristic absorption peaks are then labeled and compared with standard reference spectra to analyze the chemical structure of the cryogel.

Thermal stability: The thermal stability of the cryogel samples was evaluated using differential scanning calorimetry (DSC) and thermogravimetric analysis (TGA). Prior to measurement, the cryogels were thoroughly dried under vacuum at 50 °C for 24 h to remove residual moisture. For DSC analysis, approximately 5–10 mg of the dried cryogel was weighed and sealed in a standard aluminum pan. The sample was then heated from 0 °C to 700 °C at a constant heating rate of 10 °C/min under a continuous nitrogen flow of 100 mL/min to prevent oxidation. An empty aluminum pan was used as a reference. The DSC thermograms were recorded to observe endothermic and exothermic transitions, providing insight into the thermal transitions and stability of the material.

For the TGA analysis, a comparable amount (5–10 mg) of dried cryogel was placed in a platinum crucible and heated under identical conditions (0 °C to 700 °C; 10 °C/min; nitrogen atmosphere at 100 mL/min). The mass loss of the sample was continuously recorded as a function of temperature. The resulting TGA curves were used to determine the decomposition temperatures, thermal degradation behavior, and the residual mass at high temperature, thereby assessing the overall thermal stability of the cryogel.

SEM imaging: The cryogel samples were first freeze-dried at −80 °C for 48 h to preserve their internal microstructure. The dried cryogels were then sectioned into slices approximately 3–5 mm in thickness. Each section was mounted on an aluminum stub using conductive carbon tape and sputter-coated with a ~10 nm layer of gold to enhance surface conductivity. SEM imaging was performed using a field-emission scanning electron microscope operated at an accelerating voltage of 5–10 kV. Micrographs were acquired at various magnifications to characterize the surface morphology and porous architecture of the cryogel.

Swelling performance: Dry the cryogel at 60 °C to constant weight and record m_0_. Immerse in deionized water until equilibrium swelling, blot gently to remove surface water, and record m_1_. Lightly press the swollen gel to expel free water and record m_2_. Use these masses to assess swelling degree and water-retention capacity [126].

The swelling degree of the cryogel was calculated using the following formula:

(3) $$Swelling\;Degree=\frac{m_{1}-m_{0}}{m_{0}}\times 100\%$$

The macroporosity of the cryogel was calculated using the following equation:

(4) $$Macroporosity=\frac{m_{1}-m_{2}}{m_{1}}\times 100\%$$

Mechanical testing: Fully swollen cryogel samples were gently pressed to remove free water and then cut into cylindrical specimens (13 mm diameter × 13 mm height). A texture analyzer was used to perform cyclic compression tests at 70% strain. The test parameters were set as follows: crosshead speed = 50 mm/min, dwell time =2.0 s, and initial preload = 0.15 N. The load applied by the probe and sample displacement were recorded throughout each cycle. Key parameters for each cycle—including target displacement and the displacements at the start and end of each cycle—were saved for analysis.

Pore size distribution: Freeze-dried cryogel samples were analyzed using a mercury intrusion porosimeter. The applied pressure range was 0–100 psi, and the mercury intrusion rate was maintained at 0.1 mL/min.

Swelling behavior test: Cryogel samples were dried at 60 °C to constant weight; this dry mass was recorded as md. Samples were then immersed in deionized water to swell fully. At predetermined intervals, samples were removed, surface water gently blotted, and the swollen mass (mt) recorded. This procedure was repeated until the mass stabilized, yielding the equilibrium swollen mass (me). The swelling ratio was calculated from the dry mass (md) and equilibrium mass (me).

### 4.5. Other Assay Methods

Protein concentrations were determined using the BCA Protein Assay Kit (Sigma-Aldrich, Catalog No. B9643), following the manufacturer’s instructions. The absorbance was measured at 562 nm using a microplate reader, and bovine serum albumin (BSA) was used to generate the standard calibration curve, as described in Clerici et al. [127].

Endotoxin levels in extracted lipopolysaccharide samples were quantified using the ToxinSensor™ Chromogenic Limulus Amebocyte Lysate (LAL) Endotoxin Assay Kit (GenScript, Catalog No. L00350, Piscataway, NJ, USA), according to the manufacturer’s protocol. Absorbance at 545 nm was measured, and endotoxin concentrations were calculated from a standard curve constructed using known concentrations of *E. coli* O111:B4 endotoxin, in line with methods previously described by Flórez et al. [128].

### 4.6. Data Analysis

Unless otherwise specified, all experiments were performed in triplicate, and data are presented as mean ± standard deviation. Graphs were generated using Origin 8.0 and GraphPad Prism 10. One-way analysis of variance (ANOVA) followed by Tukey’s post hoc test was used to evaluate statistical significance. Differences were considered statistically significant at *p* < 0.05 (95% confidence level) and highly significant at *p* < 0.01 (99% confidence level).

## Figures and Tables

**Table 1:** Effects of different second monomers on cryogels.

Cryogel	Swelling Ratio (%)	Porosity (%)	Activated-Epoxy-Group Density (μmol/g)
CG(AM)	9.53	73.29	22.31
CG(HEMA-co-AM)	10.6	73.02	26.23
CG(SA-co-AM)	/	/	/
CG(PVA-co-AM)	8.33	70.23	23.54

**Table 2:** Effects of polymer concentration on cryogels.

Total Polymer Concentration (*w*/*v*)	Swelling Ratio (%)	Porosity (%)	Activated-Epoxy-Group Density (μmol/g)
5	/	/	/
6	9.83	71.92	26.87
7	11.65	72.32	28.78
8	10.35	72.23	28.72
9	9.12	71.88	28.81

**Table 3:** Effects of monomer ratio on cryogels.

Monomer Ratio (AM:HEMA)	Swelling Ratio (%)	Porosity (%)	Activated-Epoxy-Group Density (μmol/g)
1:3	12.5	76.53	25.75
1:2	12.58	76.32	26.8
1:1	13.25	75.64	28.32
2:1	13.55	77.64	30.45
3:1	12.42	78.88	27.4

**Table 4:** Pore size parameters of cryogels.

Cryogel	Volume Density (g/mL)	Skeleton Density (g/mL)	Average Pore Size (nm)	Median Pore Size (nm)	Total Pore Volume (mL/g)
CG(HEMA-co-AM)	1.0746	52,671.73	63,129.74	46,725.19	8.8149
CG(AM)	1.2131	25,388.31	25,811.63	23,946.56	7.6106

**Table 5:** Swelling kinetic parameters.

Parameter	CG(AM)	CG(HEMA-co-AM)	CG(SA-co-AM)	CG(HEMA-co-AM)
*k*	0.6746	0.4595	0.11506	0.60217
*n*	0.14312	0.28277	0.69595	0.18013
R^2^	0.99233	0.97995	0.87671	0.9915

**Table 6:** Mechanical properties of cryogels.

Cryogel	Cohesion	Elasticity	Adhesion (N)
CG(AM)	0.9	0.93	1.89
CG(HEMA-co-AM)	0.9	0.97	1.81
CG(SA-co-AM)	0.8	0.87	2.12
CG(PVA-co-AM)	0.8	0.83	1.32

**Table 7:** Effects of epoxy activator on cryogels.

Type of Activator	Activated-Epoxy-Group Density (μmol/g)
Epichlorohydrin	30.35
1,4-Butanediol diglycidyl ether	30.76

**Table 8:** Adsorption kinetic parameters and Langmuir equation parameters.

**CG(HEMA-co-AM)@ECH@PMB**	Equation	Iteration	R^2^
Pseudo-second-order kinetics	$\frac{t}{q_{t}}=\frac{1}{k{q_{e}}^{2}}+\frac{1}{q_{e}}t$	6	0.9904
Langmuir	$\frac{C_{eq}}{Q}=\frac{K_{d}}{Q_{max}}+\frac{C_{eq}}{Q_{max}}$	8	0.9907

**Table 9:** Mass spectrometry parameters of PMB.

Analyte	Retention Time	Precursor Ion	Product Ion	Fragmentor Voltage	Collision Energy
PMB	1 min	402.1 m/z	396.2 m/z	130 V	10 V

## Data Availability

Data are contained within the article.

## Abbreviations

The following abbreviations are used in this manuscript:

BSA	Bovine serum albumin
Hb	Hemoglobin
HSA	Human serum albumin
OVA	Ovalbumin
LYS	Lysozyme
